# A Case of Primary Biliary Cholangitis in a Patient With Multiple Sclerosis

**DOI:** 10.7759/cureus.63812

**Published:** 2024-07-04

**Authors:** Tigran Kakhktsyan, Mesrop Aleksanyan, Talar Acob, Knkush Hakobyan

**Affiliations:** 1 Internal Medicine, Capital Health Regional Medical Center, Trenton, USA

**Keywords:** autoimmune conditions, primary biliary cholangitis, primary biliary cirrhosis, pbc, multiple sclerosis

## Abstract

Multiple sclerosis (MS) is a chronic inflammatory disease that causes demyelination in the brain and spinal cord, leading to significant neurological disability in young adults. Patients with MS are predisposed to other autoimmune disorders, though the co-occurrence of MS and primary biliary cholangitis (PBC) is rare. PBC is an autoimmune liver disease that affects bile ducts, leading to cholestasis and liver cirrhosis, predominantly in women aged over 40 years. We report the case of an 81-year-old woman with a history of MS and hypertension, bedridden for 10 years, who was admitted with a severe sacral ulcer and bacteremia. During hospitalization, she developed persistent itching, and elevated liver enzymes were detected. Imaging ruled out cholecystitis but revealed a large gallstone and hepatomegaly. Elevated M2 antimitochondrial antibodies confirmed PBC. The patient was treated with ursodeoxycholic acid, leading to symptom improvement. This case highlights the necessity for a thorough evaluation of autoimmune comorbidities in patients with MS and suggests a potential genetic and environmental link between MS and PBC. Further research is needed to explore this association and improve treatment strategies.

## Introduction

Multiple sclerosis (MS) is a chronic inflammatory disease characterized by the presence of multiple areas of demyelination in the brain's white matter and the spinal cord [1]. It stands as the primary cause of chronic neurological disability among young adults in developed countries [2]. Patients with MS are more likely to develop other autoimmune disorders than those in the general population. These autoimmune comorbidities include uveitis, inflammatory bowel disease (IBD), Bell's palsy, Guillain-Barré syndrome, bullous pemphigoid, type 1 diabetes, ulcerative colitis, and autoimmune thyroiditis [3,4]. It is essential to recognize that the likelihood of autoimmune comorbidities differs across various diseases in individuals with MS. Previously referred to as primary biliary cirrhosis, primary biliary cholangitis (PBC) is a rare autoimmune disease that targets the bile ducts within the liver. This disease causes inflammation and scarring of the biliary ducts, leading to cholestasis and eventually progressing to liver cirrhosis [5,6]. It is noteworthy that PBC primarily affects females, occurring about nine times more frequently in women than in men. Additionally, this condition tends to manifest in individuals who are 40 years of age or older [7]. Although many patients remain asymptomatic upon diagnosis, some may experience symptoms such as fatigue or itching. The association between MS and PBC is rare, with only a limited number of cases reported [8]. In this study, we review a case of new-onset PBC in an elderly woman with MS.

## Case presentation

An 81-year-old woman with a past medical history of MS, resulting in residual neurological impairment, had been bedridden for the past ten years. She also had a history of hypertension. The patient was admitted to the hospital due to a severe stage 4 sacral ulcer leading to bacteremia. Her MS had been diagnosed approximately 20 years ago.

During her hospitalization, the patient began experiencing widespread itching, which had initially been attributed to a medication side effect. Despite being given Benadryl, the itching persisted, and applying skin moisturizers yielded no improvement. On the ninth day of her hospital stay, the patient's liver enzymes started to elevate. The alkaline phosphatase (ALP) levels increased to 368 Units/L, alanine transaminase (ALT) levels rose to 103 Units/L, and aspartate transaminase (AST) levels increased to 81 Units/L. As a result, gamma-glutamyl transferase (GGT) levels were assessed and found to be elevated at 158 Units/L (Table 1).

**Table 1:** Laboratory findings. Ab, antibody; IgM, immunoglobulin M

Test	Result	Normal range
Alkaline phosphatase (ALP)	368 Units/L	44-147 Units/L
Alanine transaminase (ALT)	103 Units/L	7-56 Units/L
Aspartate transaminase (AST)	81 Units/L	10-40 Units/L
Gamma-glutamyl transferase (GGT)	158 Units/L	9-48 Units/L
M2 antimitochondrial antibody	123.2 Units	0-20 Units
Antinuclear antibodies (ANA)	Negative	Negative
Hepatitis A IgM Ab	Negative	Negative
Hepatitis Bs Ag	Negative	Negative
Hepatitis C Ab	Negative	Negative
Hepatitis B core IgM Ab	Negative	Negative

**Figure 1:**
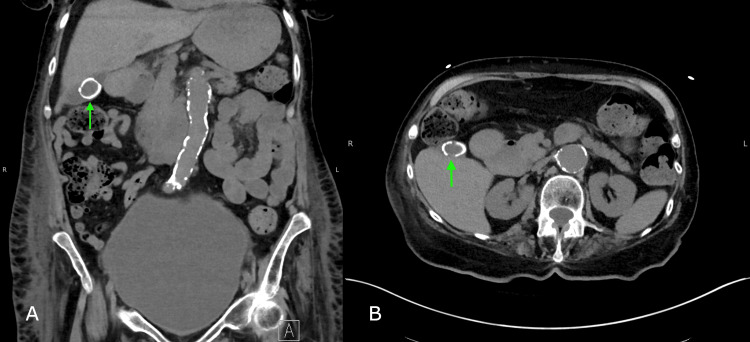
Computed tomography (CT) scan of the abdomen and pelvis without intravenous contrast. (A) Coronal plane and (B) axial plane Green Arrow: a 2.1 cm gallbladder stone.

Upon physical examination, the patient exhibited no jaundice in the sclerae, and her skin appeared normal. The abdomen was soft and not distended. The liver edge was palpable approximately 2 cm below the costal margin. There was no tenderness in any abdominal quadrant, and Murphy's sign was negative. Peritoneal signs were also absent. The patient's urine and stool showed normal coloring. Initially, the medical team investigated the possibility of acute hepatitis and cholecystitis. However, tests for hepatitis A, B, and C all returned negative results. An ultrasound scan of the liver and gallbladder was inconclusive, and a subsequent computed tomography (CT) scan of the abdomen and pelvis revealed a large calcified gallstone without any signs of gallbladder wall thickening or pericholecystic fluid. A repeat CT scan of the abdomen and pelvis confirmed the presence of a 2.1 cm gallstone (Figure 1) but ruled out cholecystitis.

**Figure 2:**
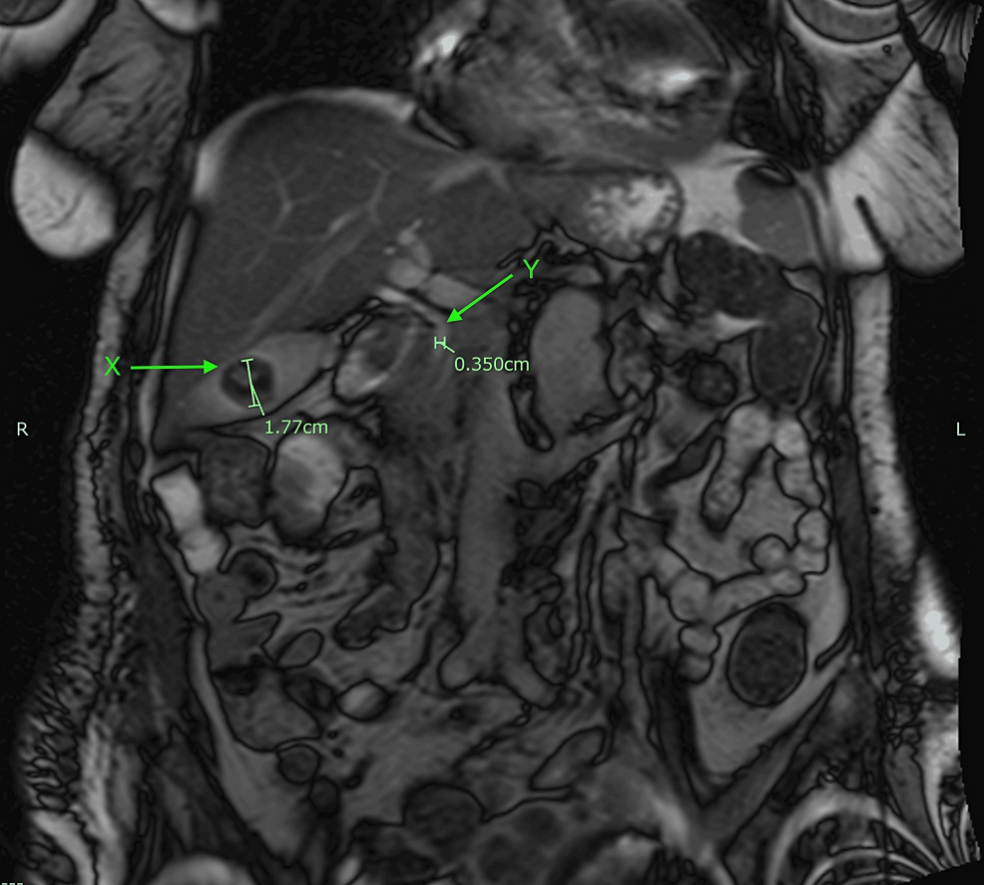
Magnetic resonance cholangiopancreatography (MRCP). Green arrow (X): a 17.7 mm stone in the gallbladder. Green arrow (Y): a 3.5 mm common bile duct.

Furthermore, a magnetic resonance cholangiopancreatography (MRCP) showed no dilation of the intrahepatic or extrahepatic biliary ducts and no thickening of the gallbladder wall or pericholecystic fluid. The MRCP did reveal a 17.7 mm gallstone (Figure 2) and hepatomegaly. The common bile duct had a normal diameter of 3.5 mm (Figure 2), and no strictures or choledocholithiasis were observed.

An M2 antimitochondrial antibody test revealed an elevated level of 123.2 units (normal range 0-20 units), while antinuclear antibodies (ANA) were negative (Table 1). A gastroenterologist was involved in the care of the patient after M2 antimitochondrial antibodies came back elevated. Based on these findings, a diagnosis of primary biliary cholangitis was established. The patient was started on ursodeoxycholic acid, which inhibits apoptosis of hepatocytes and stimulate biliary secretion at a dosage of 300 mg three times daily, leading to a gradual improvement in her symptoms, and eventually, she was discharged from the hospital.

## Discussion

Autoimmune disorders, such as MS, often occur alongside other autoimmune conditions like systemic lupus erythematosus, rheumatoid arthritis, chronic active hepatitis, type 1 diabetes mellitus, uveitis, pemphigus, psoriasis, inflammatory bowel disease, and autoimmune thyroiditis [3,4,9]. These disorders stem from abnormalities in the immune system, where the body fails to recognize its own antigens, leading to an atypical immune response against them. Both humoral and cell-mediated immune irregularities have been observed, and in some cases, multiple autoimmune diseases can coexist within a single individual, suggesting a shared underlying mechanism. Approximately 25% of patients with autoimmune diseases have a predisposition to developing additional autoimmune conditions [9].

While some studies have suggested a connection between MS and certain gastrointestinal disorders such as inflammatory bowel disease, specifically ulcerative colitis, the relationship between MS and PBC remains poorly documented [9]. Both MS and PBC are chronic autoimmune diseases characterized by chronic inflammation and the activation of immune responses that target affected tissues. In the case of MS, the humoral immune system is triggered by CD4+ and CD8+ T-lymphocytes, which can cause damage to myelin and nerves in the central nervous system (CNS). Furthermore, the involvement of both antibody-dependent and antibody-independent B cells has been observed [10,11]. In PBC, T-cells infiltrate liver tissue, leading to an inflammatory response. These activated T-cells release cytokines and other immune mediators that contribute to the destruction of bile ducts [12,13]. It is believed that the interaction between B-cells and T-cells plays a vital role in sustaining the autoimmune response in PBC. However, further research is needed to fully understand the association between MS and PBC.

Shared genetic susceptibility and common environmental risk factors have been put forth as potential explanations for the concurrent occurrence of autoimmune diseases [14].

In a study, it was discovered that there exists a notable genetic association between MS and primary biliary cirrhosis. The study specifically investigated fourteen genetic variants linked to MS and observed that higher MS-polygenic risk scores (PRS) were indicative of an elevated risk for primary biliary cirrhosis, and conversely, an increased risk of MS was associated with PBC. The PBC-PRS, which resulted in a twofold increase in primary biliary cirrhosis risk, also raised the risk of MS by 29%. Conversely, the MS-PRS, doubling the risk of MS, led to an 81% higher risk of PBC [15].

The overlap of MS and PBC is rarely reported in the literature. In a case series report, three younger women (27, 51, and 52 years old) were presented with different scenarios. The 52-year-old developed MS after being diagnosed with PBC three years earlier. She initially responded to treatment with methylprednisolone and glatiramer acetate but experienced MS relapses within the following year. The 27-year-old suffered severe neurological symptoms, including vertigo, ataxia, and weakness, and was diagnosed with MS. Two months later, she was diagnosed with PBC. Despite treatment, she died within a year. The third case presents a 51-year-old woman with a history of uveitis and Behcet disease, who developed pruritus and jaundice indicative of PBC eight years ago. She later presented with vertigo and paresthesia, leading to a brain MRI that revealed multiple lesions characteristic of MS [9].

While the presence of MS alongside other uncommon autoimmune diseases can have implications for treatment approaches, documenting such cases can contribute to early detection and improved outcomes. The prognosis of PBC has significantly improved with the use of ursodeoxycholic acid, and individuals with mild PBC can expect a normal life expectancy [16]. Research indicates that patients who are diagnosed with mild PBC and demonstrate a favorable biochemical response to ursodeoxycholic acid treatment have a more favorable prognosis.

## Conclusions

While it is well known that patients with autoimmune diseases, such as MS are more prone to have another autoimmune disease, there are only a few cases reported of concurrent MS and PBC. Performing a thorough and appropriate evaluation is essential to rule out other autoimmune diseases in patients diagnosed with MS, particularly when there is a significant level of suspicion. Our case illustrates a successful and safe example of diagnosing and treating PBC with symptom improvement. Nevertheless, it is crucial to conduct further comprehensive research to establish the potential association between MS and PBC and to establish a treatment strategy for better outcomes.
